# Re-emphasizing the concept of adequacy of intraoperative assessment of the axillary sentinel lymph nodes for identifying nodal positivity during breast cancer surgery

**DOI:** 10.1186/1477-7819-5-18

**Published:** 2007-02-09

**Authors:** Stephen P Povoski, Donn C Young, Michael J Walker, William E Carson, Lisa D Yee, Doreen M Agnese, William B Farrar

**Affiliations:** 1 Section of Surgical Oncology of the Department of Surgery, Arthur G. James Cancer Hospital and Richard J. Solove Research Institute and Comprehensive Cancer Center, The Ohio State University, Columbus, Ohio, 43210, USA; 2 Center for Biostatistics, Arthur G. James Cancer Hospital and Richard J. Solove Research Institute and Comprehensive Cancer Center, The Ohio State University, Columbus, Ohio, 43210, USA

## Abstract

**Background:**

Although sentinel lymph node (SLN) biopsy is a standard of care for the evaluation of the axillary lymph nodes during breast cancer surgery, a substantial degree of variation exists among individual surgeons as to what represents an adequate assessment. The aim of the current study was to assess when metastatic disease was first identified within consecutively harvested SLN candidates for invasive breast cancers demonstrating a positive SLN.

**Methods:**

We retrospectively analyzed a series of 400 breast cancers from a recently published prospective randomized clinical trial. A combined radiocolloid and blue dye technique was used. All potential SLN candidates, containing counts of at least 10% of the hottest SLN and/or containing blue dye, were harvested and were consecutively numbered in the order of the decreasing level of counts (with the hottest SLN representing SLN #1).

**Results:**

Among 371 invasive breast cancers, a SLN was identified within 353 cases (95%). Mean number of SLNs identified was 2.5 (range, 1 to 9), with a single SLN identified in 104 (29%) cases, two identified in 110 (31%), three identified in 73 (21%), four identified in 35 (10%), five identified in 16 (5%), and six or more identified in 15 (4%). A positive SLN was found in 104 (29%) cases. SLN #1 was the first positive SLN in 86 (83%). SLN #2 was the first positive SLN in 15 (14%). SLN #3, SLN #4, and SLN #5 were the first positive SLN in one case (1%) each. A positive SLN was found in 18% (19/104) of cases when a single SLN was identified, as compared to in 34% (85/249) when two or more SLNs were identified (P = 0.003).

**Conclusion:**

The accurate and optimal assessment of the axilla during breast cancer surgery requires persistence and diligence for attempting to identify all potential SLN candidates in order to avoid failing to recognize a positive SLN. The scenario in which only a single negative SLN candidate is intraoperatively identified is one that should raise some concern to the operating surgeon.

## Background

The application of sentinel lymph node (SLN) mapping and biopsy to the surgical staging of the axilla during breast cancer surgery has become a universally accepted standard of practice [1]. Despite widespread use of this technology, very little emphasis appears to be placed on defining the adequacy of intraoperative assessment of the axilla during breast cancer surgery. Defining the concept of an adequate intraoperative assessment of the axilla should be based upon any given principle that minimizes the risk of potentially failing to recognize a positive SLN and thereby assures accurate axillary staging during breast cancer surgery. The major determinant in this intraoperative assessment process clearly relates to be the number of SLNs harvested. In this regard, we have attempted to address this concept by retrospectively analyzing a series of 400 breast cancers from a recently published prospective randomized clinical trial [2] and determining when metastatic disease was first identified within consecutively harvested SLNs from those invasive breast cancers demonstrating a positive SLN.

## Methods

We retrospectively analyzed a series of 400 breast cancers (including 392 patients with unilateral breast cancer and 4 patients with synchronous bilateral breast cancers) from a recently published prospective randomized clinical trial that was conducted to evaluate the efficacy of the intradermal, intraparenchymal, and subareolar injection routes for the administration of radiocolloid [2]. A detailed description of patient eligibility, study design, and study execution for this recently completed prospective randomized clinical trial has been previously reported [2]. This previously reported series of 400 breast cancers included 371 cases of invasive breast cancer and 29 cases of ductal carcinoma in situ (DCIS). For the purposes of this current report, only the invasive breast cancers were included for analyses.

On the day of surgery, all patients were injected with approximately 400 μCi of ^99 m^Tc-sulfur colloid (filtered to 0.20 or 0.22 microns/micrometers) by either the intradermal, intraparenchymal, or subareolar injection route.

Intraoperatively, patients were injected intraparenchymally with approximately 5 mL of blue dye. This was generally 1% isosulfan blue dye. However, 1% methylene blue dye was used during periods of time when 1% isosulfan blue dye was not commercially available. One patient inadvertently did not receive an intraparenchyal blue dye injection. All SLNs were identified by using a hand-held gamma probe detection unit to detect radiocolloid ("hot") uptake and using visual inspection to detect blue dye ("blue") uptake. One of two hand-held gamma probe detection units was available for use by the operating surgeon, and consisted of either the Navigator GPS unit (Tyco Healthcare, Mansfield, Massachusetts) or the Neoprobe neo2000 unit (Neoprobe Corporation, Dublin, Ohio). As previously reported [2], a SLN was defined as any lymph node that was either "hot" and "blue", "hot" only, or "blue" only. A "hot" SLN was defined as any lymph node which contained a level of radioactivity that was 10% or greater of the total level of radioactivity found in the "hottest" SLN. A "blue" SLN was defined as any lymph node which visibly stained blue, had a contiguous blue-stained afferent lymphatic channel, or both. All potential SLN candidates, containing radiocolloid counts of at least 10% of the hottest SLN and/or containing blue dye, were harvested and were consecutively numbered in the order of the decreasing level of radiocolloid counts. In this regard, the hottest SLN was defined as SLN #1, regardless of the arbitrary order in which it was harvested along with the other SLNs.

Frozen section analysis with hematoxylin-eosin staining was generally performed on all submitted SLN candidates at the time of surgery. An axillary lymph node dissection was performed at the time of the SLN biopsy if any SLNs were found to contain malignant cells on frozen section analysis. Subsequently, all submitted SLNs were serially sectioned and stained with hematoxylin-eosin and immunohistochemical staining for cytokeratins (AE1:AE3) was performed.

For categorical variable univariate comparisons, either Pearson chi-square test or Fisher exact test was utilized. Continuous variables were expressed as means (± standard deviation) and/or median (range). For continuous variable univariate comparisons, one-way analysis of variance (ANOVA) was utilized. All reported univariate P-values were two-sided. All univariate P-values determined to be 0.05 or less were determined to be significant. Multivariate logistic regression analysis was then performed on all variables with a univariate P-value of 0.100 or less for the determination of possible independent predictors. The software program SPSS 14.0 for Windows (SPSS, Inc., Chicago, Illinois) was used for all statistical analyses.

## Results

Among 371 invasive breast cancers, a SLN was identified within 353 cases (95%). This included a "hot" SLN in 95% (352/371) of cases and a "blue" SLN in 68% (252/370) of cases. Of the 352 patients injected with both ^99 m^Tc-sulfur colloid and IP blue dye, the SLNs were both "hot" and "blue" in 71% (251/352), "hot" only in 28% (100/352), and "blue" only in 0.3% (1/352). Axillary concordance (defined as the proportion of cases in which both ^99 m^Tc-sulfur colloid and IP blue dye localized to the same axilla, and at least one SLN was both "hot" and "blue") was seen in 97% (245/252) of cases.

The mean number of SLNs identified was 2.5 (range, 1 to 9), with a single SLN identified in 29% (104/353) of cases, two SLNs identified in 31% (110/353), three SLNs identified in 21% (73/353), four SLNs identified in 10% (35/353), five SLNs identified in 5% (16/353), and six or more SLNs identified in 4% (15/353).

A positive SLN was found in 29% (104/353) of cases. A positive SLN was found in 18% (19/104) of cases when a single SLN was identified, as compared to in 34% (85/249) of cases when two or more SLNs were identified (P = 0.003). Patient demographics, tumor characteristics, and intraoperative variables were compared for those invasive breast cancers in which one SLN was identified versus two or more SLNs were identified (Table 1). Univariate analysis revealed that greater body weight (P = 0.041), increasing breast size (P = 0.053), and more differentiated tumors (P = 0.032) were associated with a higher likelihood of identifying only a single SLN. A similar trend was seen for greater body mass index (BMI), but this did not reach statistical significance (P = 0.101) on univariate analysis. However, multivariate logistic regression analysis revealed no independent predictors of the likelihood of identifying only a single SLN.

**Table 1:** Patient demographics, tumor characteristics, and intraoperative variables of invasive breast cancers (n = 353) in which a one SLN was identified versus two or more SLNs were identified.

	One SLN identified (n = 104)	Two or more SLNs identified (n = 249)	P-value
Age (mean ± SD, years)	56 ± 11	56 ± 12	0.870
Height (mean ± SD, inches)	64 ± 3	64 ± 2	0.386
Weight (mean ± SD, pounds)	174 ± 45	165 ± 35	0.041
BMI (mean ± SD, kg/m^2^)	29.5 ± 6.9	28.3 ± 6.0	0.101
Breast size			0.053
Small	6 (6%)	25 (10%)	
Medium	31 (30%)	101 (41%)	
Large	41 (39%)	83 (33%)	
Not available	26 (25%)	40 (16%)	
Palpable tumor	53 (51%)	120 (48%)	0.635
Tumor location			0.259
UOQ	51 (49%)	142 (57%)	
LOQ	21 (20%)	33(13%)	
UIQ	20 (19%)	38 (15%)	
LIQ	9 (9%)	21 (8%)	
Central	3 (3%)	15 (6%)	
Type of diagnostic breast biopsy			0.301
Core	79 (76%)	191 (77%)	
Excisional	24 (23%)	58 (23%)	
Fine needle aspiration	1 (1%)	0 (0%)	
Pathologic T-stage			0.617
T1	68 (65%)	176 (71%)	
T2	34 (33%)	69(28%)	
T3	2 (2%)	4 (2%)	
Histopathology			0.958
Ductal	90 (87%)	216 (87%)	
Lobular	3 (3%)	18 (7%)	
Mixed Ductal-Lobular	3 (3%)	9 (4%)	
Other	8 (8%)	6 (2%)	
Histologic grade			0.032
Well-differentiated	11 (11%)	56 (23%)	
Moderately-differentiated	47 (45%)	101 (41%)	
Poorly-differentiated	46 (44%)	92 (37%)	
Estrogen receptor positive	77 (74%)	198 (80%)	0.258
Progesterone receptor positive	63 (61%)	165 (66%)	0.308
HER-2/neu positive	27 (26%)	46 (19%)	0.136
Lymphovascular invasion	24 (23%)	48 (19%)	0.419
Route of injection of ^99 m^Tc-sulfur colloid			0.428
Intradermal	33 (32%)	92 (37%)	
Intraparenchymal	38 (37%)	74 (30%)	
Subareolar	33 (32%)	83 (33%)	
Surgery performed at time of SLN biopsy			0.184
Lumpectomy	80 (77%)	169 (68%)	
Mastectomy	21 (20%)	74 (30%)	
SLN biopsy alone	3 (3%)	6 (2%)	
Time from ^99 m^Tc-sulfur colloid injection to SLN biopsy (mean ± SD, minutes)	290 ± 78	287 ± 66	0.682

BMI, body mass index; UOQ, upper outer quadrant; LOQ, lower outer quadrant; UIQ, upper inner quadrant; LIQ, lower inner quadrant; SLN, sentinel lymph node.

SLN #1 was the first positive SLN in 83% (86/104) of cases. SLN #2 was the first positive SLN in 14% (15/104), with SLN #2 containing a mean of 45% (range, 5% to 86%) of the total counts of the hottest SLN. SLN #3, SLN #4, and SLN #5 were the first positive SLN in one case (1%) each, with SLN #3, SLN #4, and SLN #5 containing 17%, 14%, and 15% of the total counts of the hottest SLN, respectively. For all SLN-positive invasive breast cancers, patient demographics, tumor characteristics, and intraoperative variables were compared for those cases in which SLN #1 was the first positive SLN versus those cases in which SLN #1 was not the first positive SLN (Table 2). Univariate analysis revealed that older patient age (P = 0.042), smaller pathologic T-stage (T1 versus T2/T3) (P = 0.056), and lobular and/or mixed ductal-lobular histopathology (P = 0.032) were associated with a higher likelihood of SLN #1 not being the first positive SLN in SLN-positive invasive breast cancers. A similar trend was seen for the absence of lymphovascular invasion, but this did not reach statistical significance (P = 0.101) on univariate analysis. However, multivariate logistic regression analysis revealed no independent predictors of the likelihood of SLN #1 not being the first positive SLN in SLN-positive invasive breast cancers.

**Table 2:** Patient demographics, tumor characteristics, and intraoperative variables of sentinel lymph node positive invasive breast cancers (n = 104) in which SLN#1 was and was not the first positive SLN.

	SLN#1 was first positive SLN (n = 86)	SLN#1 was not first positive SLN (n = 18)	P-value
Age (mean ± SD, years)	53 ± 11	59 ± 11	0.042
Height (mean ± SD, inches)	64 ± 2	64 ± 2	0.804
Weight (mean ± SD, pounds)	164 ± 31	165 ± 36	0.883
BMI (mean ± SD, kg/m^2^)	28.2 ± 5.7	28.2 ± 6.1	0.984
Breast size			0.991
Small	9 (11%)	2 (11%)	
Medium	35 (41%)	7 (39%)	
Large	30 (35%)	6 (33%)	
Not available	12 (14%)	3 (17%)	
Palpable tumor	54 (63%)	12 (67%)	0.756
Tumor location			0.352
UOQ	45 (52%)	11 (61%)	
LOQ	19 (22%)	2 (11%)	
UIQ	9 (10%)	4 (22%)	
LIQ	5 (6%)	1 (6%)	
Central	8 (9%)	0 (0%)	
Type of diagnostic breast biopsy			0.380
Core	66 (77%)	12 (67%)	
Excisional	20 (23%)	6 (33%)	
Pathologic T-stage			0.056
T1	41 (48%)	14 (78%)	
T2	40 (47%)	3 (17%)	
T3	5 (6%)	1 (6%)	
Histopathology			0.032
Ductal	79 (92%)	13 (72%)	
Lobular	4 (5%)	2 (11%)	
Mixed Ductal-Lobular	2 (2%)	3 (17%)	
Other	1 (1%)	0 (0%)	
Histologic grade			0.584
Well-differentiated	12 (14%)	4 (22%)	
Moderately-differentiated	31 (36%)	7 (39%)	
Poorly-differentiated	43 (50%)	7 (39%)	
Estrogen receptor positive	67 (78%)	15 (83%)	0.758
Progesterone receptor positive	55 (64%)	12 (67%)	0.827
HER-2/neu positive	23 (27%)	3 (17%)	0.551
Lymphovascular invasion	37 (43%)	4 (22%)	0.101
Route of injection of ^99 m^Tc-sulfur colloid			0.450
Intradermal	34 (40%)	10 (56%)	
Intraparenchymal	24 (28%)	4 (22%)	
Subareolar	28 (33%)	4 (22%)	
Surgery performed at time of SLN biopsy			0.789
Lumpectomy	49 (57%)	10 (56%)	
Mastectomy	35 (41%)	8 (44%)	
SLN biopsy alone	2 (2%)	0 (0%)	
Time from ^99 m^Tc-sulfur colloid injection to SLN biopsy (mean ± SD, minutes)	278 ± 57	283 ± 56	0.705

BMI, body mass index; UOQ, upper outer quadrant; LOQ, lower outer quadrant; UIQ, upper inner quadrant; LIQ, lower inner quadrant; SLN, sentinel lymph node.

Table 3 shows the classification of metastatic disease within the first positive SLN for cases of invasive breast cancers in which SLN #1 was the first positive SLN versus cases in which SLN #1 was not the first positive SLN. The distribution of macrometastatic disease (> 2 mm) and micrometastatic disease (≤ 2 mm, but > 0.2 mm) were not statistically significantly different in the group in which SLN #1 was the first positive SLN versus the group in which SLN #1 was not the first positive SLN (P = 0.605 for macrometastatic disease and P = 0.759 for micrometastatic disease, respectively). However, submicrometastatic disease (≤ 0.2 mm) showed a statistically nonsignificant (P = 0.095) trend toward being more frequently seen in the group in which SLN #1 was not the first positive SLN. In the same regard, Table 4 shows the specific estimated size (in centimeters) of metastatic disease within the first positive SLN for cases of invasive breast cancer based on which of the consecutively numbered SLNs was first involved. Although one-way ANOVA of the data in Table 4 appears to show a similar trend toward decreasing size of metastatic disease with increasing consecutively numbered first positive SLNs, analogous to that found in Table 3, this trend in Table 4 was also not statistically significant (P = 0.104). Therefore, the data in Tables 3 and 4 do not clearly demonstrate a significant relationship between the volume of metastatic disease found within a given SLN and the likelihood that the hottest versus non-hottest SLN would be the first SLN to contain metastatic disease.

**Table 3:** Classification of metastatic disease within first positive SLN based on whether SLN#1 was and was not the first positive SLN for cases of invasive breast cancer (n = 104).

	SLN#1 was first positive SLN (n = 86)	SLN#1 was not first positive SLN (n = 18)	P-value
Macrometastatic (> 2 mm)	58 (67%)	11 (61%)	0.605
Micrometastatic (≤ 2 mm, but > 0.2 mm)	21 (24%)	3 (17%)	0.759
Submicrometastatic (≤ 0.2 mm)	7 (8%)	4 (22%)	0.095

SLN, sentinel lymph node.

**Table 4:** Estimated size (in centimeters) of metastatic disease within the first positive SLN for cases of invasive breast cancer (n = 104) based on which consecutively numbered SLN was first involved.

First SLN containing metastatic disease	Mean size ± SD	Median size (range)
SLN #1 (n = 86)	0.721 ± 0.691	0.500 (range, 0.005–3.000)
SLN #2 (n = 15)	0.471 ± 0.440	0.400 (range, 0.020–1.500)
SLN #3 (n = 1)	0.700	0.700
SLN #4 (n = 1)	0.200	0.200
SLN #5 (n = 1)	0.017	0.017
SLN #2, #3, #4, #5 (n = 18)	0.443 ± 0.442	0.400 (range, 0.017–1.500)

SLN, sentinel lymph node.

## Discussion

Despite the vast body of existing literature on SLN technology for breast cancer (with over 2700 publications to date), it is surprising how few papers have specifically addressed the concept of how the number of SLN candidates harvested during breast cancer surgery influences the adequacy of intraoperative assessment of the axilla [3-15]. To their credit, two well-respected groups (the University of Louisville Breast Cancer SLN Multiinstitutional Study group [3,4,12] and the Memorial Sloan-Kettering Cancer Center group[5,6]) have previously attempted to most clearly bring this concept to our attention. However, despite their attempts to bring this concept to the forefront of the surgical literature, there is an apparent ongoing lack of awareness among the general surgical community as to its importance. This has clearly resulted in a paucity of formalized published guidelines to help individual surgeons optimize the accurate assessment of the axilla during breast cancer surgery. In the current study, we have attempted to again re-emphasize this concept of what represents an optimal assessment of the axilla by retrospectively analyzing our recently published prospective randomized SLN clinical trial [2].

In the current study, while 83% of cases had the positive SLN identified as the hottest SLN, 17% of cases had the positive SLN identified as the second, third, fourth, or fifth hottest SLN. Similarly, the SLN was positive in a significantly greater frequency of cases when two or more SLNs were found as compared to when a single SLN was found (34% versus 18%, P = 0.003). Taken together, these results should raise significant concern with any patient scenario in which only a single negative SLN candidate is identified intraoperatively.

This general concern with regards to a single SLN candidate has previously been raised by the University of Louisville Breast Cancer SLN Multiinstitutional Study group [3,4,12] and the Memorial Sloan-Kettering Cancer Center group [5,6]. The most compelling aspect of this concern relates specifically to the results reported by the University of Louisville Breast Cancer SLN Multiinstitutional Study group [3,4,12] for patients who underwent SLN biopsy and a concomitant confirmatory axillary lymph node dissection. In 2001, Wong et al [4] showed a false negative rate of 14.3% in patients who had one SLN harvested as compared to 4.3% in patients who had two or more SLNs harvested (P = 0.0004). Identically, in 2005, Martin et al [12] reproduced those same results, showing a false negative rate of 13.7% in patients who had one SLN harvested as compared to 5.4% in patients who had two or more SLNs harvested (P < 0.0001). These two studies [4,12], as well as others [3,5,6,9,13,14], clearly emphasize the importance of removal of all lymph nodes containing radioisotope counts, regardless of relative magnitude of those radioisotope counts in comparison to the hottest SLN, in order to achieve maximum accuracy for the SLN biopsy procedure. In contrast, several other previous studies have not as strongly endorsed this contention [7,8,10,11,15], with some authors suggesting a focused histopathologic examination of the first two SLNs on frozen section analysis [7] or the first three SLNs on permanent analysis [11], and with other authors suggesting the placement of a limit on the "optimal" number of SLNs to harvest [8,10,15]. Nevertheless, it appears intrinsic and implied within all the above cited studies [3-15] that the identification of a single negative SLN impacts negatively on the accurate assessment of the axilla during breast cancer surgery.

While those previous studies [3-6,9,12-14] supporting the importance of harvesting all radioisotope-containing lymph nodes have emphasized this concept from the "more is better" standpoint, conversely they have not emphasized this concept from the "less is worse" standpoint. Although potentially redundant, we propose that one should also emphasize the "less is worse" point of view since there are as yet no formal criteria for defining an adequate evaluation of the axilla during breast cancer surgery. In this regard, this has perpetuated a sense of limited awareness among the general surgery community. This limitation in awareness is clearly reflected within the substantial degree of variation that exists among individual surgeons with regards to their SLN biopsy methodology [16]. Most specifically, we are referring to those surgeons who routinely identify only a single SLN candidate in the vast majority of their breast cancer cases. From the above body of evidence, those particular patients in which only a single negative SLN is intraoperatively obtained are theoretically at greater risk for inadequate assessment for the axilla, and if harboring unrecognized metastatic disease are ultimately at risk for not receiving what would be the most appropriate adjuvant therapy for their actual, but unrecognized, disease stage. So, it is that particular practice of routinely only harvesting a single SLN candidate that raises our intraoperative trepidation. In this particular situation, we strongly advise persistence and diligence with the gamma probe for attempting to identify any additional radioisotope-containing SLN candidates in order to potentially avoid failing to recognize a positive SLN and to assure that the axilla is accurately staged. Additionally, as previously emphasized by others, this recommendation for a meticulous intraoperative search should also include a thorough examination of the axilla for any blue dye-containing lymph nodes and any clinically suspicious palpable lymph nodes [3,6,17].

## Conclusion

The accurate and optimal assessment of the axilla during breast cancer surgery requires persistence and diligence for attempting to identify all potential SLN candidates in order to avoid failing to recognize a positive SLN. The scenario in which only a single negative SLN candidate is intraoperatively identified is one that should raise some concern to the operating surgeon.

## Abbreviations

BMI, body mass index; UOQ, upper outer quadrant; LOQ, lower outer quadrant; UIQ, upper inner quadrant; LIQ, lower inner quadrant; SLN, sentinel lymph node

## Competing interests

The author(s) declare that they have no competing interests.

## Authors' contributions

**SPP **was the principle investigator that supervised and conducted the original prospective randomized clinical trial [2] from which the data of this current manuscript were obtained. He designed the current study, collected the data, and performed all data analyses. He organized, wrote, and edited all aspects of this manuscript. **DCY **was the Biostatistician on this study who participated in study design, data analyses, and read and approved the final version of this manuscript. **MJW**, **WEC**, **LDY**, **DMA**, and **WBF **contributed patients to the original prospective randomized clinical trial [2] from which the data of this current manuscript were obtained. They all read and approved the final version of this manuscript.
